# BPA and Altered Airway Cells: Association Seen in Rhesus Macaques after Third-Trimester Exposure

**DOI:** 10.1289/ehp.121-a254

**Published:** 2013-08-01

**Authors:** Lindsey Konkel

**Affiliations:** Lindsey Konkel is a Worcester, MA–based journalist who reports on science, health, and the environment. She writes frequently for *Environmental Health News* and *The Daily Climate*.

Prenatal bisphenol A (BPA) exposure has been shown to alter the development of reproductive organs in animal models,^1^ although the impacts on development of other organ systems remain largely unknown. Researchers at the University of California, Davis, now report in *EHP* that BPA exposure late in gestation alters airway cell development in rhesus macaques.^2^

Previous studies have associated BPA exposure with an experimental model of asthma in mice.^3^ Epidemiological studies have found evidence of an association between prenatal BPA exposure and wheeze in young children,^4^ and between postnatal exposure and childhood asthma.^5^ “This study sheds light on the possible mechanisms by which BPA may affect lung health,” says Kathleen Donohue, an assistant professor of medicine at Columbia University. Donohue was not involved in the current study.

BPA exposure is widespread. One report from the National Health and Nutrition Examination Survey found that more than 90% of urine samples collected from U.S. males and females over age 6 years contained detectable levels of the chemical.^6^

For the current study, pregnant rhesus macaques received BPA via subcutaneous implant for 50 days during gestational days 50–100 or 100–150 (roughly comparable to the second and third trimesters, respectively, in humans). Control macaques received a corn oil implant or ate corn oil–treated fruit. Treatment groups included at least 6 animals, with histopathologic analyses conducted on smaller subgroups.

“Our goal was to model constant serum levels of BPA that have been measured in humans,” says first author Laura Van Winkle, a toxicologist at the university. “Lung development patterns and cellular abundance in the airways of these animals match humans much more closely than rodent models.”

At the end of each group’s exposure period the researchers collected fetal airway tissue samples. They used only female fetuses, as the study originated from a project designed to examine the effects of BPA on female reproductive development.

They found that BPA exposure in late pregnancy was associated with increased expression of secretory proteins in fetal tissue. The cells that produce these proteins mature late in gestation; the proteins themselves—Clara cell secretory protein (CCSP) and the mucins MUC5AC and MUC5B—are key components of airway mucous secretions.^2^

Expression of the *Muc5B* gene was approximately six times higher at 150 days’ gestation in fetuses whose mothers were exposed to BPA late in pregnancy compared with those whose mothers received no BPA exposure. Expression of the *Muc5AC* gene also was increased. Histological staining of the lung tissue indicated there were more mucous cells in the airway epithelium of exposed fetuses and suggested an increase in the amount of mucous production (however, the researchers did not have a large enough sample size to run statistical analyses).^2^

**Figure:**
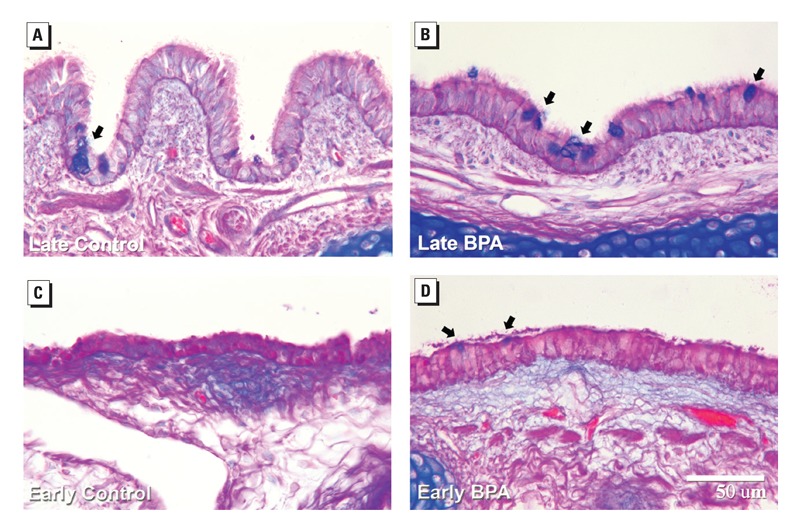
Late-pregnancy exposure to BPA (B) was associated with a higher number of mucous cells in the airway epithelium of exposed fetuses, compared with exposure in mid-pregnancy (D) and controls (A and C). Van Winkel et al./DOI:10.1289/ehp.1206064

There were no significant changes in protein expression after exposure during mid-pregnancy, suggesting that late pregnancy may be a critical period in which BPA exposure may alter airway cell development. This critical window may also apply to human exposures to BPA in the third trimester of pregnancy due to similarities in the timing of cellular development and airway structure between rhesus macaques and humans.^2^

“Taken together with earlier studies in humans, this study in monkeys is important because it is another link in the chain of evidence [potentially] connecting BPA exposure to lung disease,” says Donohue.

An increase in mucous cell abundance is one of the hallmarks of asthma, the authors report. However, they say, the clinical relevance of this particular study remains to be seen. The researchers examined only fetal airway tissue samples, and it is unknown whether an increase in mucous cell abundance would have resulted in airway disease after birth.

The potential effects of environmental estrogens such as BPA on lung development also are not well known, says Van Winkle. Although estrogen is known to increase the expression of *MUC5B* in cultured airway epithelium cells from humans,^7^ and lung tissue does have estrogen receptors,^8^ it’s not clear how this affects the development of secretory proteins.

Recent experimental studies have suggested that BPA may also disrupt fetal development through epigenetic and nonestrogenic pathways.^9^^,^^10^ Van Winkle suspects a combination of hormonal and nonhormonal effects may be at play. “A third possibility is that BPA affects something else entirely in the body that in turn alters lung development,” she says.
